# Transcriptional Profiling of Human Peripheral Blood Mononuclear Cells Stimulated by *Mycobacterium tuberculosis* PPE57 Identifies Characteristic Genes Associated With Type I Interferon Signaling

**DOI:** 10.3389/fcimb.2021.716809

**Published:** 2021-08-19

**Authors:** Fanli Yi, Jing Hu, Xiaoyan Zhu, Yue Wang, Qiuju Yu, Jing Deng, Xuedong Huang, Ying Ma, Yi Xie

**Affiliations:** Department of Laboratory Medicine, West China Hospital, Sichuan University, Chengdu, China

**Keywords:** *Mycobacterium tuberculosis*, PPE57, peripheral blood mononuclear cell, RNA sequencing, type I interferon signaling

## Abstract

Proline-glutamic acid (PE)- and proline-proline-glutamic acid (PPE)-containing proteins are exclusive to *Mycobacterium tuberculosis* (*MTB*), the leading cause of tuberculosis (TB). In this study, we performed global transcriptome sequencing (RNA-Seq) on PPE57-stimulated peripheral blood mononuclear cells (PBMCs) and control samples to quantitatively measure the expression level of key transcripts of interest. A total of 1367 differentially expressed genes (DEGs) were observed in response to a 6 h exposure to PPE57, with 685 being up-regulated and 682 down-regulated. Immune-related gene functions and pathways associated with these genes were evaluated, revealing that the type I IFN signaling pathway was the most significantly enriched pathway in our RNA-seq dataset, with 14 DEGs identified therein including *ISG15, MX2, IRF9, IFIT3, IFIT2, OAS3, IFIT1, IFI6, OAS2, OASL, RSAD2, OAS1, IRF7*, and *MX1*. These PPE57-related transcriptomic profiles have implications for a better understanding of host global immune mechanisms underlying *MTB* infection outcomes. However, more studies regarding these DEGs and type I IFN signaling in this infectious context are necessary to more fully clarify the underlying mechanisms that arise in response to PPE57 during *MTB* infection.

## Introduction

Tuberculosis (TB) is an airborne communicable infection primarily caused by *Mycobacterium tuberculosis* (*MTB*) (Pai et al., 2016). Despite the advent of antibiotics and the *Mycobacterium Bovis bacillus* Calmette-Guérin (BCG) vaccine, millions of individuals are infected with TB each year (O'Garra et al., 2013). TB incidence is growing in part due to multidrug-resistant strains and to high rates of human immunodeficiency virus (HIV) co-infection (Sayes et al., 2012). In addition, the BCG vaccine is thought to be less effective as a means of maintaining long-term immunity in adults as compared to children (Yang et al., 2016), underscoring the importance of developing novel immunological approaches to preventing or treating this disease.

PE/PPE proteins containing highly conserved N-terminal Pro-Glu (PE) or Pro-Pro-Glu (PPE) motifs comprise approximately 10% of the *MTB* genome (Cole et al., 1998), and are key targets for vaccine development. The 99 and 69 characterized *pe* and *ppe* genes, respectively, contain extensive repetitive domains and a high GC content (Brennan, 2017). Comparative genomic analyses of mycobacterial species have shown that these PE/PPE proteins are primarily present in slower-growing mycobacterial pathogens (Cole, 2002), with the majority being proteins expressed on the cell surface or released into the extracellular space (Sampson, 2011; Mitra et al., 2017). These proteins are thought to be related to mycobacterial virulence, growth, and modulation or evasion of host immune responses (Vordermeier et al., 2012; Saini et al., 2016). PPE57 (*Rv3425*) is a PPE family protein encoded within the open reading frame in RD1 of *M. tuberculosis* H37Rv (Behr et al., 1999; Xu et al., 2015). Previous studies have indicated that *Rv3425* was able to increase the production of IFN-γ in TB patients than in the healthy controls (Zhang et al., 2007; Chen et al., 2009). PPE57 can interact with Toll-like receptor 2 (TLR2) on macrophages to induce their activation and can promote type 1 helper T cell (Th1) polarization, while recombinant BCG-PPE57 can enhance protective immunity against *MTB* infection (Xu et al., 2015). Owing to its well-documented effects on human immune cells, PPE57 may thus alter the transcriptional profiles of exposed human immune cells.

Peripheral blood mononuclear cells (PBMCs) collected *via* venipuncture contain all of the key immune cells necessary for the coordination of *MTB* defenses. PBMCs can serve as sensitive biomarkers capable of differentiation between active TB infections and healthy individuals (Qian et al., 2016; Roe et al., 2016). RNA sequencing (RNA-seq) is a high-throughput technique to assess the full transcriptome of samples to identify gene expression in a manner more comprehensive than traditional microarray analyses (Stark et al., 2019). Blood transcriptomic profiling is frequently conducted to evaluate host immune responses to particular stimulants or pathogens (Wang et al., 2016; Fang et al., 2020). Herein, we employed an RNA-seq approach to identify genes that were differentially expressed in PBMCs upon stimulation with PPE57 to better understand global host-specific mechanisms related to the immune response in the context of TB infection.

## Materials and Methods

### Ethical Statement and Participant Inclusion Criteria

This study was approved by the Ethics Committee of West China Hospital, Sichuan University(reference: 2021-756). All participants provided written informed consent to participate in this study. The Science and Technology Department Project of Sichuan, China approved the present study (2020YFS0555). In total, six healthy participants (3 males, 3 females; 25 – 45 years old) of similar ethnic backgrounds were recruited for the present study. These participants did not report any prior history of recurrent infections, and were completely healthy at the time of sample collection.

### Blood Processing and *In Vitro* Stimulation

In total, 20 mL of peripheral blood was collected during the morning from each donor in a vacutainer containing EDTA-K2 as an anticoagulant (BD, America). Blood samples were processed within 2 h of isolation. PBMCs were initially isolated *via* gradient centrifugation using Lymphocyte Cell Separation Media (Stemcell, Canada) based on provided directions. Briefly, the blood was initially diluted with 2 mL of PBS, after which it was carefully layered on top of the Lymphoprep™ solution in a conical tube, followed by centrifugation for 25 min at 500 × *g*. The white interface layer was then collected, and the PBMCs therein were washed two times with RPMI-1640 (Gibco, USA), with centrifugation being conducted for 10 min at 500 × *g*. Cells were resuspended in 8 mL of RPMI-1640 containing 10% FBS (Gibco) and 1% penicillin-streptomycin (Hyclone, USA). Cells were then counted with a hemocytometer, after which approximately 10 × 10^6^ PBMCs from each donor were collected and either stimulated with PPE57 (4 µg/mL; Shanghai Gene-optimal Science & Technology Co., Ltd. China) or were left untreated for 6 h during which they were incubated at 37°C in a 5% CO_2_ incubator. All procedures were conducted in a BSL-2 laminar flow hood under sterile conditions to prevent endotoxin contamination, with all replicate samples being processed under identical conditions.

### RNA Isolation and Library Construction

TRIzol was used to isolate total RNA from treated PBMCs based on provided directions, after which a NanoDrop 2000 instrument (Thermo Scientific, USA) was used to assess RNA quantity. An Agilent 2100 Bioanalyzer (Agilent Technologies, CA, USA) was utilized to measure RNA integrity, with samples that had RNA integrity values (RIN) > 7 being used for downstream RNA-seq analyses. A TruSeq Stranded mRNA LT Sample Prep Kit (Illumina, CA, USA) was used to prepare cDNA libraries based upon provided directions. OE Biotech Co., Ltd. (Shanghai, China) performed all RNA-sequencing and bioinformatics analyses.

### RNA Sequencing and Bioinformatics Analysis

An Illumina Hiseq X Ten device was used for 150-bp paired-end sequencing for analyzed samples. Resultant raw read data in the fastq format were initially processed with Trimmomatic (Bolger et al., 2014), with low-quality reads being removed. Cleaned reads then underwent human reference genome alignment *via* hisat2 (Kim et al., 2015), and mRNA abundance was estimated by calculating FPKM values with cufflinks (Trapnell et al., 2010). HTSeq-count was used to establish gene read counts (Anders et al., 2015). Differentially expressed genes (DEGs) were detected with a DESeq (2012) R package, with P < 0.05 and FC > 2 or FC < 0.5 as the cutoff criteria for differential expression. Our raw RNA-seq data were deposited in the NCBI Sequence Read Archive (www.ncbi.nlm.nih.gov/sra/) with accession number PRJNA715906.

Hierarchical cluster analyses were conducted on identified DEGs in order to evaluate their expression patterns. Gene Set Enrichment Analysis (GSEA http://software.broadinstitute.org/gsea/index.jsp) is a computational pathway analysis tool for exploring whether a given gene set shows statistically significant, concordant differences between two biological states (Subramanian et al., 2005; Liberzon et al., 2011; Liberzon et al., 2015), with P < 0.05 as a cutoff for significance. R was used to conduct GO and KEGG pathway analyses of DEGs based upon a hypergeometric distribution, with P < 0.05 as the significance threshold. The top 100 DEGs were additionally used to construct a protein-protein interaction (PPI) network using the STRING database (https://string-db.org/), with targeted sub-networks additionally being constructed to assess the prospective functions of these DEGs.

### qRT-PCR

Gene expression was assessed *via* a qRT-PCR approach. Individual RT-PCR reactions were comprised of 0.5 μg RNA, 2 μl of 5×TransScript All-in-one SuperMix for qPCR, and 0.5 μl of gDNA Remover in 10 μl, and were amplified to generate cDNA in a GeneAmp^®^ PCR System 9700 instrument (Applied Biosystems, USA) for 15 min at 42°C, followed by 5 s at 85°C. The resultant mixture was then diluted 10-fold and stored at -20°C. Next, qRT-PCR analyses were performed with a LightCycler^®^ 480 II Real-time PCR Device (Roche, Switzerland), with each 10 μl sample containing 1 μl cDNA, 5 μl 2×PerfectStartTM Green qPCR SuperMix, 0.2 μl of each primer, and 3.6 μl of nuclease-free water. Analyses were performed with 384-well plates (Roche) with thermocycler settings of: 94°C for 30 s; 45 cycles of 94°C for 5 s, 60°C for 30 s. Samples were analyzed in triplicate, and melt curve analyses were performed to examine amplified PCR product specificity. Primers were synthesized by TsingKe Biotech according to NCBI database mRNA sequences (**Supplementary Table 1**). Relative gene expression was normalized to *ACTB* and calculated *via* the 2^-ΔΔCt^ approach.

## Results

### RNA-seq Data Summary

To begin exploring the mechanisms governing the pathogenic role of PPE57 on cells of the peripheral immune system, we performed an RNA-seq analysis (**Supplementary Table 2**). Twelve cDNA libraries were prepared and sequenced (**Table 1**). These analyses yielded 595.4 million raw reads, with 579.36 million clean reads remaining following the removal of the adaptor and low-quality sequences. Between 95.3% and 96.96% of reads in each sample were mapped to the reference genome, with 91.91% - 93.64% of these reads being uniquely mapped to the reference genome. The Q30 values corresponding to the clean reads for these 12 libraries were above 91.92%, consistent with high data reliability.

**Table 1:** Statistics for the sequenced transcriptomic data.

Sample	RawReads	CleanReads	GC Content	Q30	Mapped Reads	Unique Mapped Reads	Multiple Mapped Reads
CasePP1	51.12M	49.18M	49.69%	91.92%	47.57M (96.71%)	46.05M (93.64%)	1.51M (3.08%)
CasePP2	50.67M	49.06M	49.97%	92.37%	46.89M (95.57%)	45.35M (92.43%)	1.54M (3.14%)
CasePP3	49.14M	47.96M	49.99%	92.97%	46.30M (96.54%)	44.66M (93.13%)	1.63M (3.40%)
CasePP4	51.20M	49.96M	50.71%	92.86%	47.97M (96.01%)	46.27M (92.61%)	1.70M (3.40%)
CasePP5	49.03M	47.47M	50.91%	92.41%	45.23M (95.30%)	43.63M (91.91%)	1.61M (3.39%)
CasePP6	49.08M	48.15M	50.20%	93.58%	46.54M (96.66%)	44.80M (93.03%)	1.75M (3.62%)
Wt1	50.07M	48.80M	51.15%	93.12%	46.83M (95.96%)	45.17M (92.56%)	1.66M (3.39%)
Wt2	49.02M	47.26M	50.55%	92.12%	45.33M (95.93%)	43.80M (92.68%)	1.53M (3.25%)
Wt3	47.32M	46.00M	50.28%	92.42%	44.45M (96.63%)	42.74M (92.90%)	1.71M (3.73%)
Wt4	47.68M	46.47M	50.32%	92.92%	44.83M (96.48%)	43.29M (93.16%)	1.54M (3.32%)
Wt5	51.67M	50.63M	51.29%	93.46%	49.09M (96.96%)	47.22M (93.25%)	1.88M (3.71%)
Wt6	49.40M	48.42M	51.21%	93.58%	46.66M (96.36%)	44.91M (92.76%)	1.74M (3.60%)

CasePP and Wt respectively correspond to the stimulated and unstimulated groups, with 1-6 corresponding to six parallel replicates. M: million. GC Content corresponds to the percentage of guanine and cytosine in the cleaned reads. Q30, the percentage of nucleotides with a quality value of 30.

### Identification of DEGs

**Figure 1:**
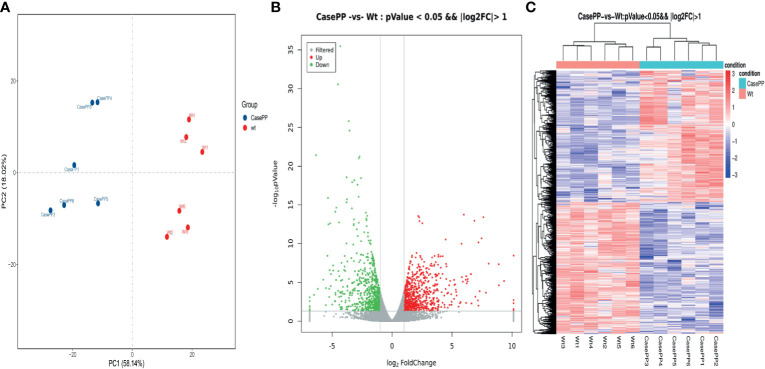
Differentially expressed genes (DEGs) identified in PBMCs stimulated with PPE57 relative to control cells. **(A)** Principal component analysis of DEG transcripts. **(B)** A volcano plot highlighting DEGs in comparisons of control and PPE57-stimulated samples (log_10_p-value *vs* log_2_FC). Green and red dots with negative and positive change values respectively correspond to downregulated and upregulated DEGs. **(C)** Hierarchical clustering of transcripts that were significantly (≥2-fold change) upregulated (red) or downregulated (blue) in PPE57-treated PBMCs, with three repeats per sample.

To investigate important gene signatures for PPE57, we performed a DEG analysis between 6 stimulated samples and 6 controls by the R package. Principal component analysis (PCA) of mRNA expression profiles in our samples revealed that identified DEGs were able to successfully differentiate unstimulated and stimulated PBMCs, consistent with these transcriptomic differences being attributable to PPE57 exposure (**Figure 1A**). In total, we identified 1367 DEGs (|log2 FC| > 1, P < 0.05), of which 685 and 682 were upregulated and downregulated in PPE57-stimulated samples, respectively (**Figure 1B** and **Supplementary Table 3**). These DEGs were visualized using a heat map (**Figure 1C**), confirming that PPE57 stimulation was associated with a significant change in PBMC gene expression profiles relative to those observed in control unstimulated samples.

### GSEA of All Genes in PPE57-Stimulated PBMCs

**Figure 2:**
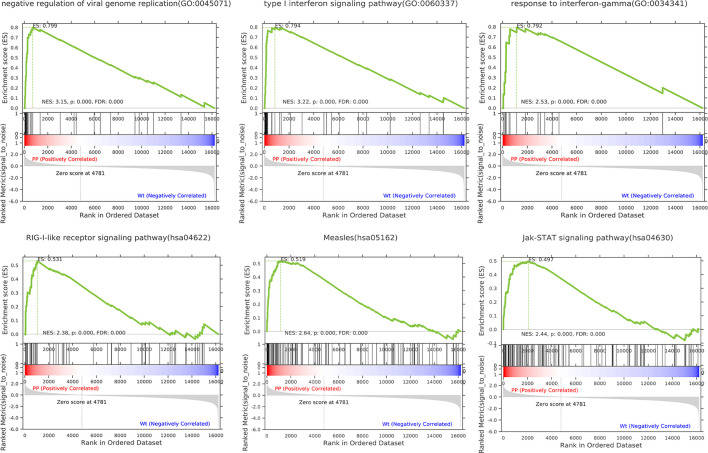
Gene set differences between PPE57-stimulated PBMCs and controls as illustrated through a gene set enrichment analysis (GSEA) approach. Enrichment plots for six GSEA pathways that were enriched in PPE57-stimulated PBMCs relative to controls. A gene set was considered to be significantly enriched at a *P* ≤ 0.05.

To more fully understand the whole-transcriptomic changes observed upon PPE57 stimulation, GSEA was next conducted. GSEA is focused on the whole gene set expression other than DEGs. As shown in **Table 2** and **Figure 2**, the results of a GSEA-based GO analysis revealed that the given gene set was significantly enriched in the negative regulation of viral genome replication, type I interferon (IFN) signaling, and response to IFN γ pathways. In this figure, higher absolute-fold normalized enrichment score (NSE) values and smaller P- and FDR values are indicative of more significant enrichment. Meanwhile, the results of a GSEA-based KEGG analysis indicated that RIG-I-like receptor signaling, measles, and JAK-STAT signaling pathways were significantly enriched. A majority of these pathways are closely related to IFN signaling. A total of 23 core genes were found to be associated with the type I IFN signaling pathway, including *MX2, MX1, IFITM1, OASL, IRF9, OAS3, OAS2, IFI6, ISG20, IFIT3, SP100, RSAD2, IRF7, IFIT1, OAS1, IFI35, ISG15, ADAR, IFIT2, BST2, IFITM3, GBP2*, and *IFI27*. The 21 core genes associated with the RIG-I-like receptor signaling pathway included *IFIH1, TRIM25, NFKB1, DHX58, DDX3X, IRF7, ISG15, DDX58, TRAF2, MAPK11, RELA, CXCL8, TBK1, IL12B, RIPK1, NFKBIA, CYLD, IL12A, TNF, AZI2*, and *TANK*. The type I IFN and RIG-I-like receptor signaling pathways are key promoters of innate antiviral immunity, and have profound impacts on adaptive immunity. These results suggested that PPE57 may thus regulate the type I IFN signaling pathway initiated by these RIG-I-like receptors (RLRs).

**Table 2:** The gene set enrichment analysis (GSEA) results for all expressed genes (Top 6 based upon enrichment score).

	Term	ES	Gene Set Size	Matched Size	Core Genes
GO	negative regulation of viral genome replication (GO:0045071)	0.79873987	33	32	MX1, IFITM1, OASL, IFIT5, OAS3, ISG20, RSAD2, IFIT1, OAS1, ISG15, ADAR, IFI16, PARP10, BST2, C19orf66, SLPI, ZC3HAV1, IFITM3, TNIP1
	type I interferon signaling pathway (GO:0060337)	0.79442268	50	39	MX2, MX1, IFITM1, OASL, IRF9, OAS3, OAS2, IFI6, ISG20, IFIT3, SP100, RSAD2, IRF7, IFIT1, OAS1, IFI35, ISG15, ADAR, IFIT2, BST2, IFITM3, GBP2, IFI27
	response to interferon-gamma (GO:0034341)	0.79216942	16	16	IFITM1, SP100, GCH1, TRIM21, BST2, C19orf66, NUB1, IFITM3, IL23R
KEGG	RIG-I-like receptor signaling pathway (hsa04622)	0.53124104	70	55	IFIH1, TRIM25, NFKB1, DHX58, DDX3X, IRF7, ISG15, DDX58, TRAF2, MAPK11, RELA, CXCL8, TBK1, IL12B, RIPK1, NFKBIA, CYLD, IL12A, TNF, AZI2, TANK
	Measles (hsa05162)	0.51850571	131	116	STAT3, JAK3, STAT5A, MX1, IL13, IFIH1, TNFAIP3, NFKB1, IRF9, OAS3, OAS2, EIF2AK2, IL2RA, IRF7, OAS1, TAB2, IL1B, ADAR, DDX58, IL1A, STAT2, FAS, IL6, STAT1, RELA, TBK1, IL12B, TNFSF10, NFKBIA, IFNG, IL4, SLAMF1, STAT5B, CCND2, IL12A, IL2RB, CCNE2, FASLG, HSPA8
	Jak-STAT signaling pathway (hsa04630)	0.49701537	162	134	STAT3, PIM1, JAK3, STAT5A, IL13, SOCS1, IRF9, IL20, SOCS3, IL2RA, IL15RA, IL11, STAT4, SOCS2, MCL1, IL7, STAT2, PTPN2, BCL2L1, IL10, IL6, CSF3, STAT1, IL12RB2, IL4R, IL23A, TSLP, IL24, IL12B, IL22, IL19, CISH, IFNG, CSF2, IL4, STAT5B, CCND2, IL12A, IL2RB, IL15, IL23R, SOS1, JAK1, IL7R, IL21R, OSM, CDKN1A, IL22RA1, BCL2, AOX1, PTPN11, PIK3R1, IL5, PDGFA, PDGFRA, IL2RG, MYC, IL2

### GO Function and KEGG Pathway Analysis of DEGs in PPE57-Stimulated PBMCs

**Figure 3:**
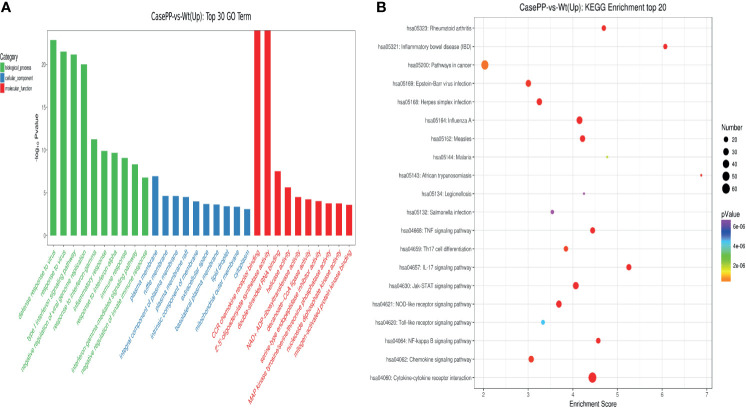
GO annotations and KEGG pathway analysis results for upregulated DEGs. **(A)** The top 30 biological process, cellular component, and molecular function GO terms are shown (P *<* 0.05; unique gene number of GO terms *>* 2). **(B)** The top 20 KEGG pathways with positive enrichment are shown in a bubble chart with enriched pathways on the *y-*axis and enrichment scores on the *x*-axis. A positive correlation between bubble size and the number of pathway-related genes was observed, with a larger pathway enrichment *P-*value being associated with an increase in the red coloration of that bubble.

To further understand the pathophysiological effects of PPE57 protein exposure in human PBMCs, we next conducted GO and KEGG pathway enrichment analysis of identified DEGs. In total, 4322 GO terms and 298 KEGG pathways were associated with these DEGs. The top 30 GO terms associated with upregulated DEGs in the biological process (BP), cellular component (CC) and molecular function (MF) categories are shown in **Figure 3A**. These BPs were primarily enriched for the following BP terms: defense response to virus, response to virus, and type I interferon signaling pathway. These DEGs were additionally enriched for CC terms including plasma membrane, ruffle membrane, and integral component of the plasma membrane, and for MF terms including CCR chemokine receptor binding, 2’-5’-oligoadenylate synthetase activity, and double-stranded RNA binding. These upregulated DEGs were associated with KEGG pathways including the cytokine-cytokine receptor interaction, influenza A, Jak-STAT signaling, IL-17 signaling, inflammatory bowel disease (IBD), measles, NOD-like receptor signaling, TNF signaling, and NF-κB signaling pathways (**Figure 3B**). A total of 22 DEGs including *ADAR, IFIT3, OAS1, OAS2, SP100, MX1, OASL, IFIT1, ISG20, IFI35, IFIT2, IRF7, IFI6, MX2, IFITM1, GBP2, BST2, ISG15, IRF9, OAS3, IFITM3*, and *RSAD2* were associated with the type I IFN signaling pathway, which is related to PPE57 pathogenic activity. Together, these results offer novel insights into the pathogenic role of PPE57 in the context of *MTB* infection.

### PPI Network Construction

**Figure 4:**
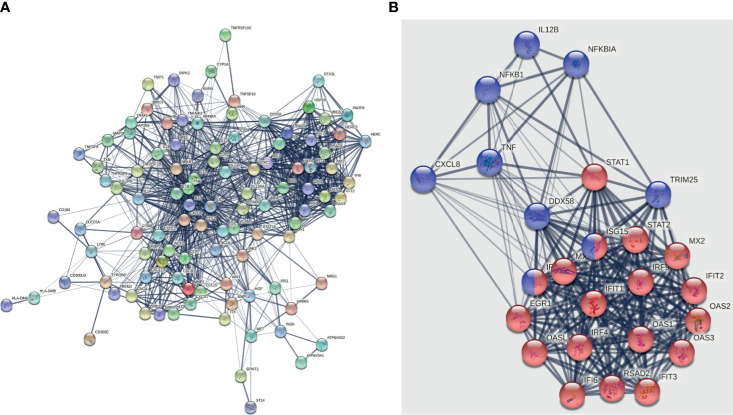
PPI networks and modules incorporating the top 100 DEGs from PPE57-stimulated PBMCs. **(A)** PPI network. **(B)** Module. The DEGs associated with the type I IFN signaling pathway are marked with red circles. DEGs associated with the RIG-I-like receptor signaling pathway are marked with blue circles.

A PPI network containing 104 nodes and 1113 edges was next constructed to assess potential interactions among the top 100 of these DEGs (**Figure 4A**). This analysis led to the identification of 18 type I interferon (IFN) signaling pathway-related DEGs including *STAT1, RSAD2, MX2, STAT2, MX1, ISG15, IFIT1, OASL, IRF9, OAS1, IFIT2, IFIT3, IRF4, IRF7, EGR1, OAS3, OAS2*, and *IFI6* (**Figure 4B**), thus suggesting that PPE57 exposure can induce type I IFN signaling in exposed PBMCs. In addition, RIG-I-like receptor signaling pathway-related DEGs including *CXCL8, TNF, NFKB1, DDX58, ISG15, NFKBIA, IL12B, TRIM25*, and *IRF7* were identified in this PPI network (**Figure 4B**), with both of these pathways being prominent among the top 100 DEGs.

### Functional Analysis of the Type I Interferon Signaling Pathway

**Figure 5:**
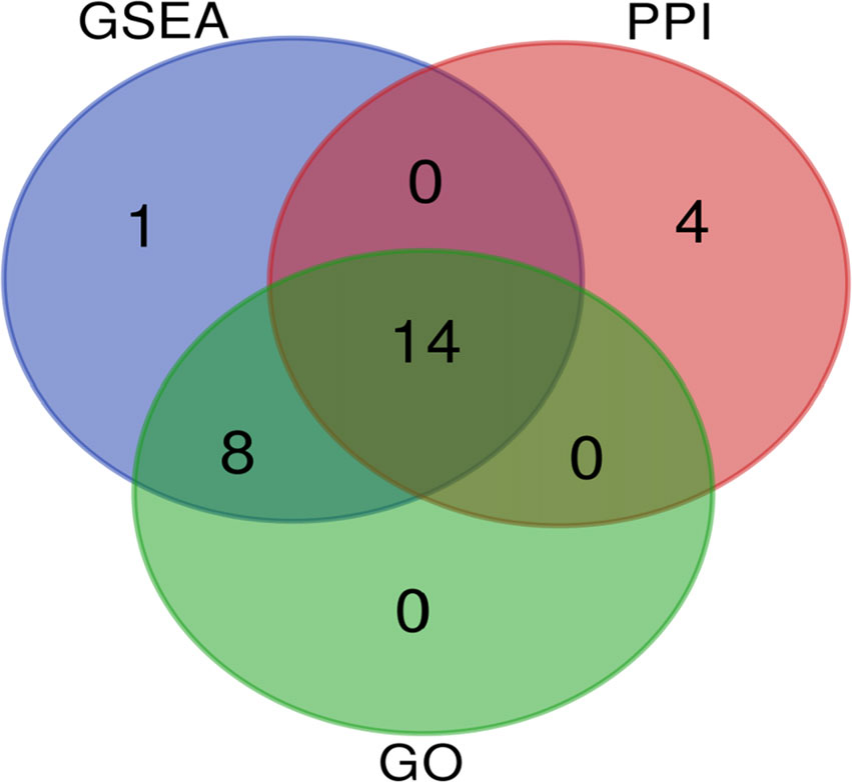
Venn diagram demonstrating the number of common overlapping genes in GSEA, GO, and PPI analyses. These overlapping genes were related to the type I IFN signaling pathway. Genes analyzed *via* GSEA, GO, and PPI approaches are denoted in blue, green, and red circles, respectively. In total, 14 genes overlapped across all three of these analyses.

In total, 27 related DEGs were found in the type I IFN signaling pathway, all of them were analyzed by Wayne analysis within three bioinformatic methods (GSEA, GO and PPI network) (**Table 3** and **Figure 5**). Specifically, *ISG15, MX2, IRF9, IFIT3, IFIT2, OAS3, IFIT1, IFI6, OAS2, OASL, RSAD2, OAS1, IRF7*, and *MX1* were 14 DEGs that shared among three bioinformatic methods and were all upregulated upon PPE57 exposure.

**Table 3:** Overlap of type I IFN signaling pathway-related genes among GO, GSEA, and PPI analysis results.

Names	Total	Elements
GO GSEA PPI	14	ISG15, MX2, IRF9, IFIT3, IFIT2, OAS3, IFIT1, IFI6, OAS2, OASL, RSAD2, OAS1, IRF7, MX1
GO GSEA	8	IFI35, ADAR, ISG20, IFITM1, SP100, GBP2, IFITM3, BST2
GSEA	1	IFI27
PPI	4	IRF4, EGR1, STAT2, STAT1

### RNA-seq Validation

**Figure 6:**
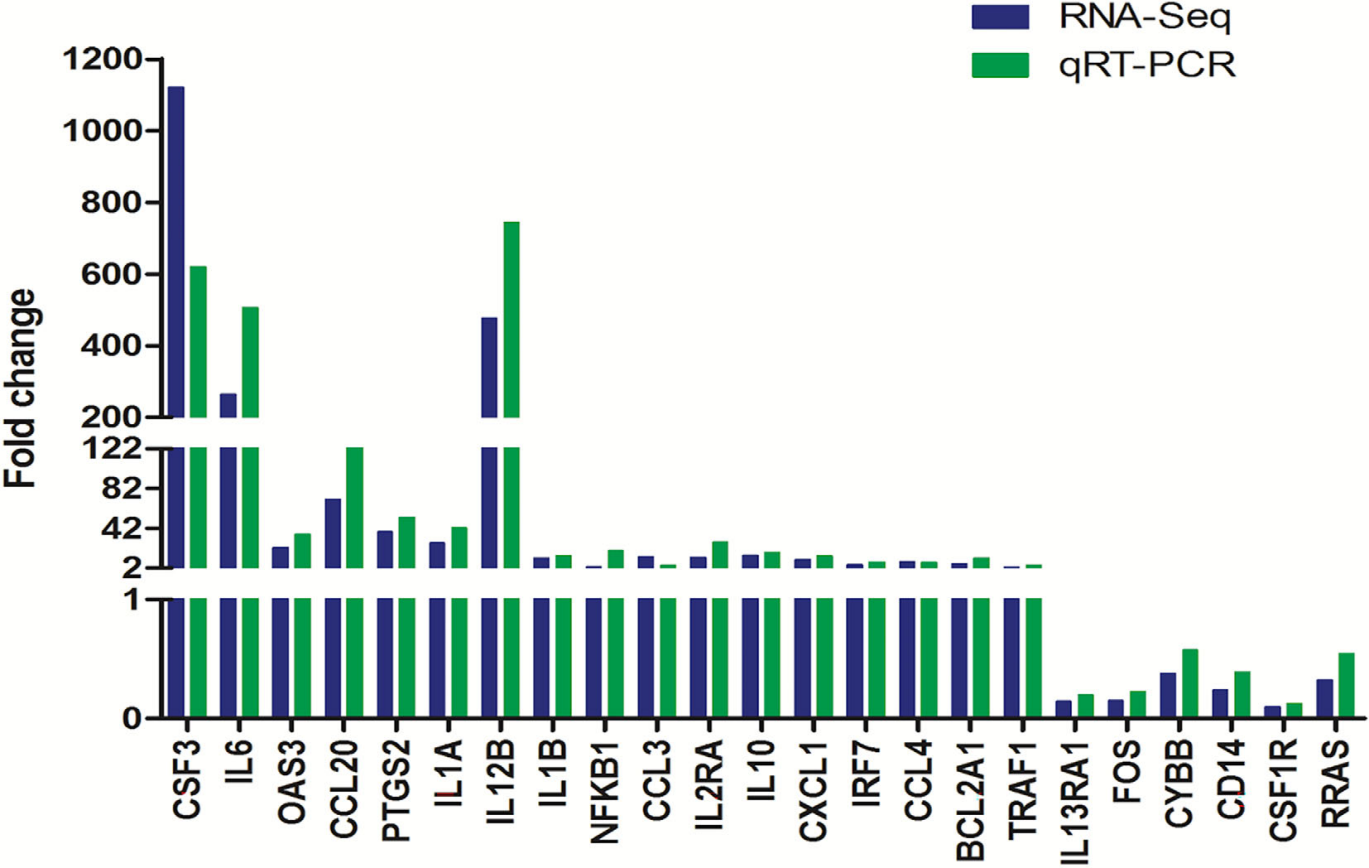
Validation of transcriptomic sequencing results by real-time qPCR.

To verify the accuracy of our RNA-seq analyses, we next selected 17 upregulated DEGs and 6 downregulated DEGs for qPCR-based validation. We found that the expression levels of these genes were comparable with what was observed in our RNA-seq analyses with a correlation coefficient of 0.85, thus confirming the accuracy and credibility of our transcriptomic results (**Figure 6**).

## Discussion

PE/PPE family proteins are thought to serve as key mediators of *MTB* pathogenicity and immunogenicity (Sampson, 2011), yet the mechanisms whereby they affect host cells remain incompletely understood. A comprehensive analysis is thus essential in order to fully elucidate the complex responses of the host immune system to these mycobacterial antigens. As such, transcriptomic and bioinformatic analyses are of value as they offer a thorough understanding of the host defense mechanisms and immune evasion strategies associated with particular infections (Zhao et al., 2017; Wang et al., 2019; Fang et al., 2020). Several studies have explored the immune effect of PPE57 exposure (Zhang et al., 2007; Wang et al., 2008; Xu et al., 2015), but further work is needed to clarify host-pathogen interactions in this context. Relative to studies of ESAT-6/CFP-10, little is known regarding the biological impact of PPE57 exposure in PBMCs. Therefore, RNA-seq was performed in this study to understand the mechanisms underlying such interactions in host PBMCs.

A total of 1367 DEGs were identified in PBMCs following PPE57 stimulation. These DEGs were further subjected to bioinformatic analyses that seek to explore the pathways/mechanisms whereby they may regulate responses to this mycobacterial protein. Overall, we discovered that changes in the DEGs expression enriched in immune-related pathways including the type I IFN signaling pathway. Indeed, this signaling pathway was the most significantly enriched in our RNA-seq dataset, with 14 DEGs identified therein including *ISG15, MX2, IRF9, IFIT3, IFIT2, OAS3, IFIT1, IFI6, OAS2, OASL, RSAD2, OAS1, IRF7*, and *MX1*.

Type I IFN production can drive the upregulation and activation of a range of well-characterized antiviral genes, which stimulate or suppress immune function in a manner that can be either protective or detrimental, depending on the context in which it occurs. Type I IFN signaling is also against some bacteria, such as *Escherichia coli*, *pneumococci*, and *group B streptococci* (Mancuso et al., 2007). In this study, GO terms related to IFNs were significantly enriched in PBMCs following PPE57 stimulation, including the type I IFN signaling and response to IFNγ pathways. Type I IFN signaling was considered to be of particular importance, given that recent transcriptional analyses have highlighted a potentially deleterious role for type I IFNs in TB (Moreira-Teixeira et al., 2018). In a recent study conducted by Zhang et al. (2021), type I IFN signaling was identified as a critical mediator of Mtb-induced macrophage cell death, and it is one of the first specific mechanisms of *Mtb*-infected macrophage death to have been identified (Zhang et al., 2021). Yoshida et al. (1988) reported that type I IFNs were able to disrupt macrophage activation through type II IFN (IFN-γ), which is required for the control of TB in humans (Yoshida et al., 1988; Jouanguy et al., 1997). *In vitro* studies of the *MTB* infection of bone marrow-derived macrophages have revealed that type I IFN signaling is essential for the early production of inducible nitric oxide synthase (Shi et al., 2005), which is critical for defending against *MTB*. In clinical trials, type I IFNs have also been shown to be beneficial against pulmonary TB (Giosué et al., 1998). Therefore, it is difficult to define a clear role for this cytokine in the context of *MTB* infection despite its importance in many other immunological settings. A previous study suggested that PPE57 may be able to enhance protective responses against *MTB* by including macrophage activation through the mitogen-activated protein kinase (MAPK) and nuclear factor κB (NF-κB) signaling pathways (Xu et al., 2015). In our study, PPE57 was able to stimulate type I IFN signaling, which may, in turn, be linked to *MTB* virulence and increased host susceptibility. A detailed examination of the type I IFN signaling pathway as it pertains to PPE57 may thus contribute to the design of host-directed therapies against *MTB* infection, although further validation will be required to confirm these results.

The type I IFN pathway is activated by the binding of this cytokine to the type I IFN receptor (IFNAR1 and IFNAR2) and the subsequent activation of the JAK/STAT signaling pathway, leading to the induction of about 300 interferon-stimulated genes (ISGs). Several of these ISGs have been found to be upregulated during *MTB* infection (Remoli et al., 2002; Chaussabel et al., 2003).

The expression of interferon-alpha inducible protein 6 (*IFI6*) has not been reported in the context of TB. In our study, *IFI6* was found to be upregulated during bacterial infection. *IFI6* encodes a mitochondrial ISG with immunomodulatory and anti-apoptotic activity (Porter et al., 1988). *IFI6* is mainly localized inside the nucleus and restricts HBV gene expression and replication *in vitro* and *in vivo* (Sajid et al., 2021). The anti-apoptotic activity of *IFI6* has been observed in breast cancer cells, gastric cancer cells, vascular endothelial cells, and human myeloma cells (Tahara et al., 2005; Cheriyath et al., 2007; Cheriyath et al., 2012; Qi et al., 2015). However, such pro-survival activity is also critical for *MTB* resistance in macrophages. As such, we hypothesized that PPE57 was able to induce the upregulation of *IFI6* whereupon it may play an anti-apoptotic role in the context of TB *via* regulating JAK/STAT signaling or related pathways, although further validation will be necessary to test this hypothesis.

During *MTB* infection, we found that *ISG15* was one of the most highly induced ISGs. Previous studies have shown that humans with an *ISG15* deficiency are susceptible to mycobacterial disease, and secreted *ISG15* can promote IFN-γ secretion (Bogunovic et al., 2012). *ISG15* mRNA levels have also been identified as a potentially valuable biomarker in the context of active human TB (Dos Santos et al., 2018), as *ISG15* can promote early *MTB* replication although it plays protective roles during the later stages of infection (Kimmey et al., 2017). Other ISGs with known antimicrobial roles (*RSAD2*, *IFIT1*, *IFIT2*, *IFIT3, MX1*, and *MX2*) were also highly upregulated in this study (Diamond, 2014; Helbig and Beard, 2014; Haller et al., 2015). To the best of our knowledge, the precise mechanisms by which many of these genes are upregulated during TB disease progression remain to be defined (Andreu et al., 2017). However, we have herein shown that PPE57 can promote the upregulation of these genes, and further research regarding their ability to shape host defense responses against *MTB* is warranted.

The 2’-5’-oligoadenylate synthetases3 (*OAS3*) exhibited the highest fold-change value of the 14 IFN-related DEGs in this study. The IFN-inducible gene profile includes both IFN-αβ and IFN-γ, as well *OAS1-3*, and 2’-5’-oligoadenylate synthetases-like (*OASL*), all of which can be regulated by both type I and type II IFNs (Zhou et al., 1997). As key ISGs, *OAS1, OAS2*, and *OAS3* are evolutionarily conserved and are associated with the early inflammatory response during infection (Justesen et al., 2000; Yao et al., 2019). Transcriptomic analyses of blood from patients with active TB indicate that the upregulation of *OAS1, OAS2*, and *OAS3* can differentiate between active and latent TB infection status (Berry et al., 2010; Maertzdorf et al., 2012; Ottenhoff et al., 2012). *OAS1, OAS2, OAS3*, and *OASL* expression levels are also directly linked to mycobacterial pathogenicity during the early post-infection period, as they can restrict *MTB* intracellular replication in macrophages by regulating cytokine secretion (Leisching et al., 2019; Leisching et al., 2020). As such, these ISGs may be involved in both promoting cellular survival and directly suppressing mycobacterial growth. Herein, we found that PPE57 exposure was sufficient to induce the upregulation of *OAS1-3* and *OASL* in PBMCs, potentially indicating that PPE57 may indirectly induce anti-*MTB* effects related to the cell wall and the entrance of these bacteria into macrophages (Xu et al., 2015).

PPE proteins are able to readily induce host immune responses and are effective immunogens (Brennan, 2017). PPE18, for example, has already been used in completed phase IIb clinical trials of the M72/AS01 vaccine candidate (Day et al., 2013; Tait et al., 2019), and PPE42 has been used in a phase Ia trial of the ID93/GLA-SE, a subunit vaccine candidate that has exhibited good protection in animal models and has been shown reliable safety and robust immunogenicity (Coler et al., 2018; Khoshnood et al., 2018; Kwon et al., 2019). Unlike these two PPE proteins, the understanding pertaining to PPE57 is relatively limited, prompting the present study. Rv3425 (PPE57), which is also a promising protein for the design of mycobacterial vaccines, is herein reported as an MTB antigen associated with ISG induction for the first time. Prior studies have shown that other PPE proteins such as PPE26 and PPE39 can also affect the IFN signaling pathways (Su et al., 2015; Choi et al., 2019). Specifically, PPE39 is capable of eliciting dendritic cell (DC) activation in a TLR4-dependent manner through downstream MAPK and NF-κB signaling pathways, promoting Th1-cell polarization and increased IFN-γ production (Choi et al., 2019). PPE57 can activate the MAPK and NF-κB signaling pathways *via* TLR2 signaling (Xu et al., 2015). In our study, we found that PPE57 was able to affect the MAPK, JAK/STAT, and NF-κB pathways, potentially following type I IFN pathway activation. Therefore, we hypothesized that the type I IFN pathway may be activated by the binding of PPE57 to the type I IFN receptor (IFNAR1 and IFNAR2), subsequently regulating the activation of ISGs and other signaling pathways. PPE57 was also able to influence RLR pathway signaling, which controls type I IFN signaling pathway activation (Hou et al., 2021). RLRs include RIG-I, MDA5, and LGP2, all of which play important roles in the recognition of viral RNA in the cytoplasm (Bruns and Horvath, 2014). MTB is an intracellular parasite, and it is thus possible that PPE57 may bind to RLRs to regulate type I IFN signaling. Future studies testing these possibilities through antibody blocking experiments or related approaches are warranted.

PPE26 also induces TLR2-dependent macrophage activation and IFN-γ secretion (Su et al., 2015). Host proteomic changes in response to PPE26 stimulation have previously been analyzed *via* iTRAQ subcellular quantitative proteomics (Su et al., 2015), revealing a significant degree of overlap with the differentially expressed genes identified in the present study. For example, *NFKB1*, *STAT1, STAT2*, and *TNF* were upregulated in both studies, yet *CD14* was downregulated in our study and upregulated in this prior study. *CD14* is a surface receptor preferentially expressed on monocytes/macrophages that is critical for the induction of appropriate immune responses to aerogenic MTB infections (Cubillos-Angulo et al., 2021). PPE26 can upregulate *CD14*, thereby potentially promoting a more adequate innate immune response. Interestingly, targeting *CD14* in the treatment of SARS-CoV-2-infected patients provides an opportunity to potentially inhibit multiple inflammatory responses (Martin et al., 2020). The pathogenic mechanisms of the intracellular *MTB* parasite may be similar to this virus in some ways such that PPE57 can downregulate the expression of *CD14*. PPE57 may thus indirectly lessen allergic reactions to *MTB*, although this requires further study. Overall, *MTB* PPE family proteins are known to be generally immunogenic, but they differ from one other with respect to their physiological effects. Exploring the pathogenicity and immunogenicity of each of these proteins is vital, thus prompting the present study of PPE57. Further research regarding other members of this protein family may similarly highlight novel clinical insights.

In this study, we employed PPE57-stimulated PBMCs to effectively study *MTB-specific* host immune responses while simultaneously minimizing unrelated background noise. Relative to other studies employing unstimulated whole blood samples, PPE57 stimulation was able to provoke more varied host immune responses, potentially improving our ability to resolve key transcriptional gene expression profiles of interest. However, as the results from the present study were obtained through a series of bioinformatics analyses, additional preclinical and clinical research will be essential to confirm and build upon these findings.

In conclusion, we comprehensively analyzed transcription profiles associated with PPE57 stimulation in PBMCs of healthy hosts and identified 14 genes (*ISG15, MX2, IRF9, IFIT3, IFIT2, OAS3, IFIT1, IFI6, OAS2, OASL, RSAD2, OAS1, IRF7*, and *MX1*) associated with type I IFN signaling that were enriched following such stimulation. These findings offer new insights into host global immune mechanisms underlying *MTB* infection and PPE57 exposure. However, additional functional studies are required to determine the specific roles of the identified genes in the context of TB pathogenesis. Overall, our data highlight the importance of examining whether type I IFN responses to PPE57 exposure induce protective or harmful effects for the host.

## Data Availability Statement

The datasets presented in this study can be found in online repositories. The names of the repository/repositories and accession number(s) can be found in the article/**Supplementary Material**.

## Ethics Statement

The study was approved by the Ethics Committee of West China Hospital, Sichuan University (reference: 2021-756). The participants provided their written informed consent for the publication of any potentially identifiable data included in this article.

## Author Contributions

YX, FY, and YM conceived and designed the study. FY conducted the experiments of the study. JH, XZ, YW, QY, JD, and XH contributed reagents/materials. FY analyzed the data and wrote the paper. YX and YM critically revised the manuscript. All authors contributed to the article and approved the submitted version.

## Funding

Project financial support was provided by the Science and Technology Department Project of Sichuan, China (2020YFS0555).

## Conflict of Interest

The authors declare that the research was conducted in the absence of any commercial or financial relationships that could be construed as a potential conflict of interest.

## Publisher’s Note

All claims expressed in this article are solely those of the authors and do not necessarily represent those of their affiliated organizations, or those of the publisher, the editors and the reviewers. Any product that may be evaluated in this article, or claim that may be made by its manufacturer, is not guaranteed or endorsed by the publisher.
